# Glioblastoma multiforme in a patient with neurofibromatosis type 1: a case report and review of literature

**DOI:** 10.1093/jscr/rjae517

**Published:** 2024-08-28

**Authors:** Laith A Ayasa, Sara Rahhal, Alaa Khaled Najjar, Bashar N Suboh, Mohammed Aliwaiai, Ahmad M Daqour, Izzeddin Bakri

**Affiliations:** Al Quds University Faculty of Medicine, Mount of Olives St. 26, Sheikh Jarrah, P.O. Box 22246, Jerusalem 91513, Palestine; School of Medicine, The University of Jordan, Amman, Queen Rania St. Jubeiha, P.O. Box 11942, Amman, Jordan; Department of Neurosurgery, Al-Makassed Islamic Charitable Hospital, Jerusalem 97103, Palestine; Department of Neurosurgery, Al-Makassed Islamic Charitable Hospital, Jerusalem 97103, Palestine; Department of Neurosurgery, Al-Makassed Islamic Charitable Hospital, Jerusalem 97103, Palestine; Department of Neurosurgery, Al-Makassed Islamic Charitable Hospital, Jerusalem 97103, Palestine; Department of Pathology, Al-Makassed Islamic Charitable Hospital, Jerusalem 97103, Palestine

**Keywords:** glioblastoma, neurofibromatosis type 1 glioma, adult

## Abstract

Glioblastoma multiforme (GBM) is a highly aggressive brain tumor. Individuals with neurofibromatosis type 1 (NF1) have an increased risk of developing GBM. We present a case report of a 44-year-old male with NF1 who developed GBM. NF1-associated GBM presents distinct molecular features and younger age at diagnosis compared to sporadic cases. Treatment typically follows standard protocols for GBM. Despite advancements in neuro-oncology, gaps in knowledge persist regarding NF1-associated GBM, including its prevalence, molecular mechanisms, and optimal treatment strategies. Larger studies and collaborative efforts are needed to address these gaps and enhance patient care.

## Introduction

Neurofibromatosis type 1 (NF1) is an autosomal dominant condition affecting multiple organ systems, with a prevalence of about 1 in 3000 individuals [1]. It is part of a group of conditions described under the term ‘neurofibromatosis,’ which also includes neurofibromatosis type 2 and schwannomatosis, each with distinct clinical and genetic characteristics. Various studies have explored the relationship between gene function and clinical manifestations of NF1, including tumor development. While NF1 commonly involves benign tumors like pilocytic astrocytomas, it is worth noting that the majority of gliomas arising in adults with NF1 are malignant, typically glioblastomas [2]. In our study, we report a rare case of a 44-year-old man with NF1, shedding light on complexities of this condition.

## Case presentation

In this case report, we present the clinical course of a 44-year-old male with NF1 who presented to our hospital with initial symptoms including headache, dizziness, ataxia, vomiting, nausea, and right eye blurring of vision for 2 weeks. Upon examination, the patient was conscious, alert, and oriented, with stable vital signs but demonstrated compromised vision in the right eye, an ataxic gait, and left homonymous hemianopia. Subsequent neuro-ophthalmic evaluation revealed decreased visual acuity and optic disc swelling in the left eye.

**Figure 1:**
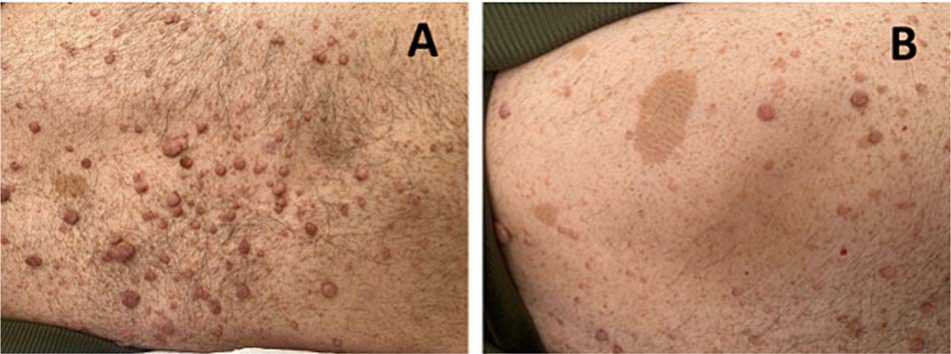
(A) shows multiple neurofibromas, which are benign nerve sheath tumors. (B) depicts café-au-lait spots observed on the patient’s skin.

The diagnosis of NF1 was supported by characteristic clinical findings, including the presence of neurofibromas and café-au-lait spots, illustrated in Fig. 1A and B, respectively.

He also had Lisch nodules, bilateral axillary freckling, and scattered neurofibromas on his trunk along with reported learning difficulties, further supporting the diagnosis of NF1.

**Figure 2:**
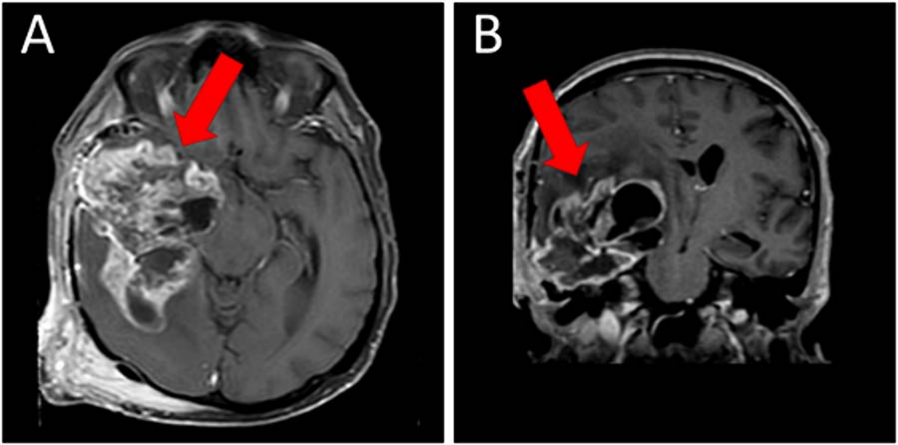
Preoperative T1WI (A) axial and (B) sagittal MRI images of a huge right temporo-parietal intra-axial lesion (indicated by arrows), which show enhancement after gadolinium injection. The axial image also shows extracranial subgaleal plexiform neurofibroma. MRI = magnetic resonance imaging, T1WI = T1-weighted images

A magnetic resonance imaging (MRI) scan showed extensive hypodense infiltrating disease in the right temporo-parietal region with associated vasogenic edema, alongside a significant space-occupying lesion (Fig. 2).

**Figure 3:**
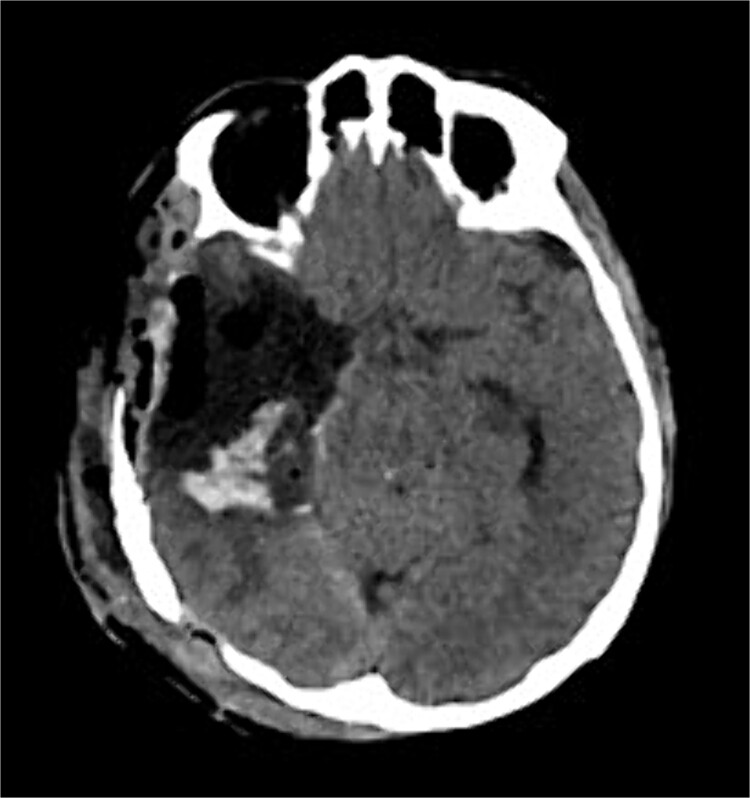
Postoperative axial CT shows postoperative changes with maximum safe resection of the lesion. CT = computed tomography.

**Figure 4:**
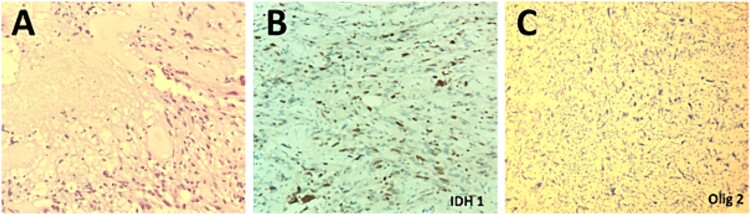
Histopathological examination of the excised lesion demonstrates characteristic features of a Glioblastoma. (A) The section shows high grade glioma with areas of necrosis and vascular proliferative changes (H&E, original magnification ×20). The neoplastic cells are negative for IDH1 immunostain (×20) (B), and positive for Olig2 immunostain (×20) (C).

The patient underwent a right temporo-parietal craniotomy for lesion resection (Fig. 3), followed by radiotherapy and temozolomide, and was transferred to the neuro intensive care unit for close monitoring. Postoperatively, the patient remained conscious, alert, and oriented, with stable vital signs, but exhibited left facial paresis, diminished vision in the right eye, and left-sided weakness. Pathological assessment confirmed a high-grade glioma, consistent with a World Health Organization grade IV tumor (Fig. 4). It revealed an infiltrative, hypercellular astrocytoma characterized by hyperchromatic, elongated nuclei with irregular contours. A significant number of mitotic figures were observed. The histopathological assessment also demonstrated geographic and pseudopalisading necrosis, accompanied by microvascular proliferation, which are hallmark features of high-grade gliomas.

The patient was kept under observation for 14 days, during which he remained in a good general condition and stable vital signs. He was advised to undergo physiotherapy sessions and was prescribed antibiotics.

The case underscores the intricate interplay between genetic predisposition, tumor biology, and treatment response, emphasizing the imperative for further research to comprehend the underlying mechanisms driving the occurrence and aggressive nature of glioblastoma in the context of NF1.

## Discussion

NF1, initially described by Friederich Daniel von Recklinghausen in 1882, represents the most common neurocutaneous syndrome. Its diagnosis relies on meeting at least two of the seven criteria established by the National Institute of Health Consensus Development Conference in 1988 (Table 1). NF1 is attributed to mutations in the NF1-gene, located on chromosome 17q11.2, a tumor-suppressor gene involved in RAS-MAPK (mitogen-activated protein kinase) signaling. Neurofibromin, the NF1-gene product, acts as a negative regulator of the p21 Ras proto-oncogene, moderating cell growth. Therefore, patients with NF1 are predisposed to a higher risk of developing various benign and malignant tumors.

**Table 1:** Diagnostic criteria for type 1 neurofibromatosis (two or more of the following criteria are required)

Six or more café-au-lait spots with the following diameter: ≥5 mm before puberty ≥15 mm after puberty Two or more neurofibromas of any type or one plexiform neurofibroma Axillary or inguinal freckling (cowden syndrome) Optic pathway glioma Two or more Lisch nodules (benign hamartomas of the iris) Typical bone lesions Sphenoid dysplasia Dysplasia or thinning of long bone cortex (pseudarthrosis) First-degree relatives with NF1

Characteristically, neurofibromas affect nearly all patients with NF1 and can progress to malignant peripheral nerve sheath tumors, contributing to poor survival rates [3]. Additionally, NF1 patients commonly develop other tumors such as pilocytic astrocytomas, gastrointestinal stromal tumors, pheochromocytomas, and juvenile myelomonocytic leukemia. Although glioblastomas are not as common in NF1 as other tumors, estimated to account for approximately 7% of cases [4], they remain a significant consideration within the spectrum of neoplasms associated with the condition. Larger studies are needed to assess glioblastoma multiforme (GBM) prevalence in NF1 patients, crucial for improving management and treatment strategies. Additionally, NF1 patients older than 10 years are estimated to have a 100-fold higher relative risk of developing brain tumors compared to individuals without NF1 [2]. However, Huttner et al., in their review of five glioblastoma patients in children with NF1, suggested that the survival of glioblastoma patients with NF1 was better than those without NF1. The median overall survival of patients with and without NF1 was 9.25 and 1.08 years, respectively [5]. NF1-associated GBMs manifest distinct molecular features, commonly lacking mutations in IDH1/2, TERT, and BRAF. Instead, they frequently exhibit the co-occurrence of p53 mutations, cooperating in malignant astrocytoma development [6, 7]. The genetic signature of somatic NF1 mutations characterizes the mesenchymal subtype of GBM, which is associated with a worse overall outcome [8]. In this report, we describe a case of GBM in a patient diagnosed with neurofibromatosis 1 (NF1). GBM represents the most prevalent primary malignant brain tumor in adults and is known for its aggressive nature, with a survival rate of less than 6% beyond 5 years of diagnosis [9]. While the majority of GBM cases occur sporadically, individuals with certain genetic disorders such as neurofibromatosis, Turcot syndrome, and Li–Fraumeni syndrome have an inherent predisposition to developing GBM [10]. It is noted that patients with NF1-associated glioblastoma are diagnosed at a significantly younger age (mean age 37 years) compared to those with sporadic glioblastoma (above 55) [9]. Our patient was diagnosed at 44 years old, which is consistent with literature. Limited data exist on glioblastomas in individuals with NF1, with few cases reported (Table 2). In most cases reported, GBM was located supratentorially with very few cases reported in the infratentorial region. In our case, the tumor was located temporo-parietally, which aligns with literature. Regarding treatment, all reported cases of glioblastoma in patients with NF, including ours, received the standard therapy for glioblastoma (gross total resection followed by fractionated radiotherapy and chemotherapy).

**Table 2:** Published cases of adult NF1 with glioblastoma

Author	Age/sex	Location	Presentation	Treatment	Follow-up	Outcome
Jeong and Yee (2014) [11]	32/M	Frontal	Progressive headache	SR + RT + CT	9 months	Resolved
Varghese and Abdul Jalal (2015) [12]	60/M	Frontal	Headache, neck pain, gait unsteadiness	SR + CT + RT		
Narasimhaiah *et al.* (2019) [13]	21/F	Fronto-parietal	Bifrontal headache, vomiting and blurring of vision	SR + CT + RT	32 months	Resolved
Cai *et al.* (2021) [7]	51/F	Temporo-parietal	Headache and left limb weakness	SR + CT + RT	13 months	resolved
Basindwah *et al.* (2022) [14]	27/M	Fronto-parietal	Severe headache, confusion, and bilateral papilledema	SR + RT + CT	2 years	Resolved
Present case	44/M	Temporo-parietal	Headache, ataxia, and right eye blurring of vision	SR + RT + CT		Resolved

M: male, F: female, SR: subtotal resection, CT: chemotherapy, RT: radiotherapy

In conclusion, NF1-associated GBM presents distinctive challenges given its unique molecular characteristics and tendency for diagnosis at a younger age. To better understand its specific molecular profiles and enhance prognosis and treatment outcomes, larger-scale studies are needed.

## Learning points

– NF1 patients may develop GBM, necessitating heightened awareness for early detection and intervention to optimize treatment outcomes, as shown in this case of a 44-year-old male.
– NF1-associated GBM presents distinctive challenges, given its unique molecular characteristics and tendency for diagnosis at a younger age.
– More research is essential to understand NF1-related GBM prevalence, molecular mechanisms, and treatment strategies.
